# Supplementary material for: Long-read sequencing of trios reveals increased germline and postzygotic mutation rates in repetitive DNA
Source: bioRxiv. 2025 Jul 19:2025.07.18.665621. Preprint. [Version 1] doi: 10.1101/2025.07.18.665621 (PMC12338747; doi:10.1101/2025.07.18.665621)
Supplement: Supplement 1 [file media-1.pdf]

SUPPLEMENT

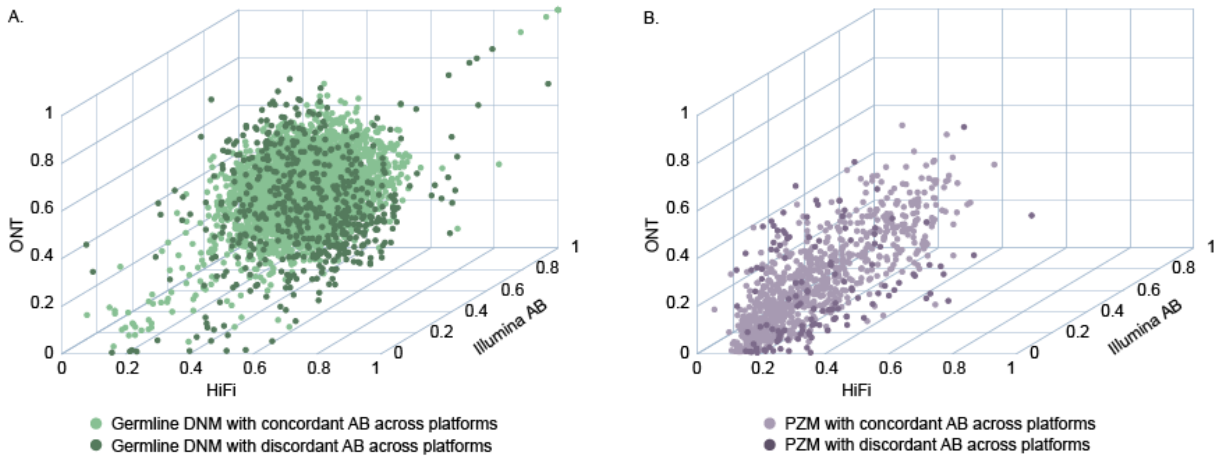

Figure S1: Allele balance scatterplots

A. Allele balance (AB) of germline DNMs across all three platforms for variants with concordant and discordant AB by chi-squared shows that most variants have AB around 0.5. B. PZMs tend to have low AB across platforms.

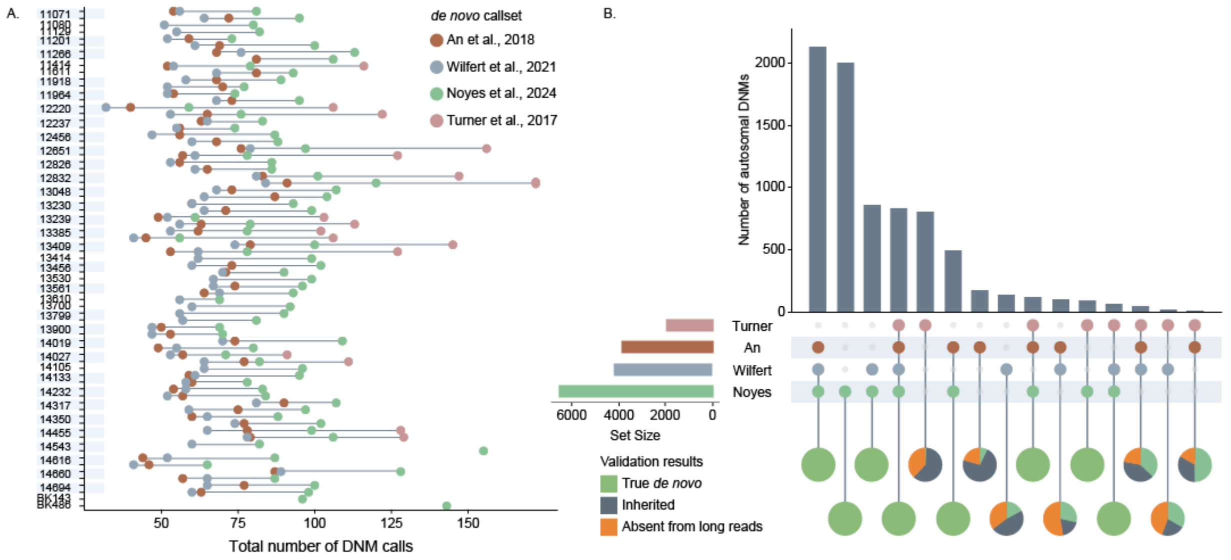

Figure S2: Comparison to other studies

A. We identified more DNMs per sample than most previous Illumina-based studies, with the exception of Turner et al. B. DNMs identified by multiple studies have the highest true positive rates, while DNMs exclusive to a single study tend to be false positives.

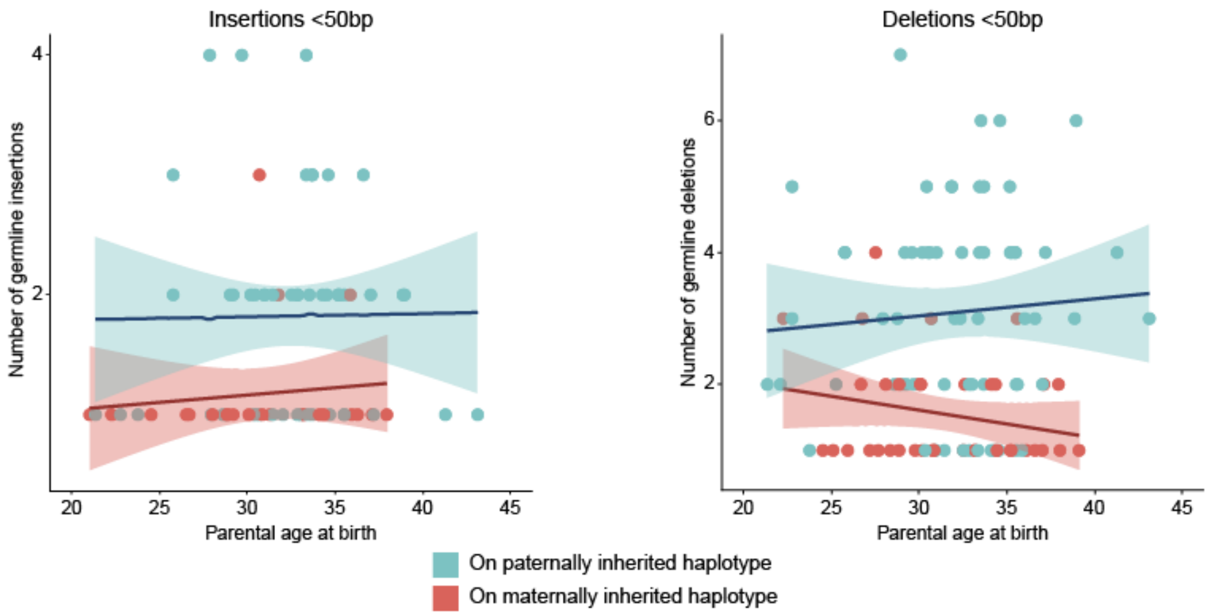

Figure S3: Indel parental age effect
Neither insertions nor deletions are significantly correlated with year of parental age.

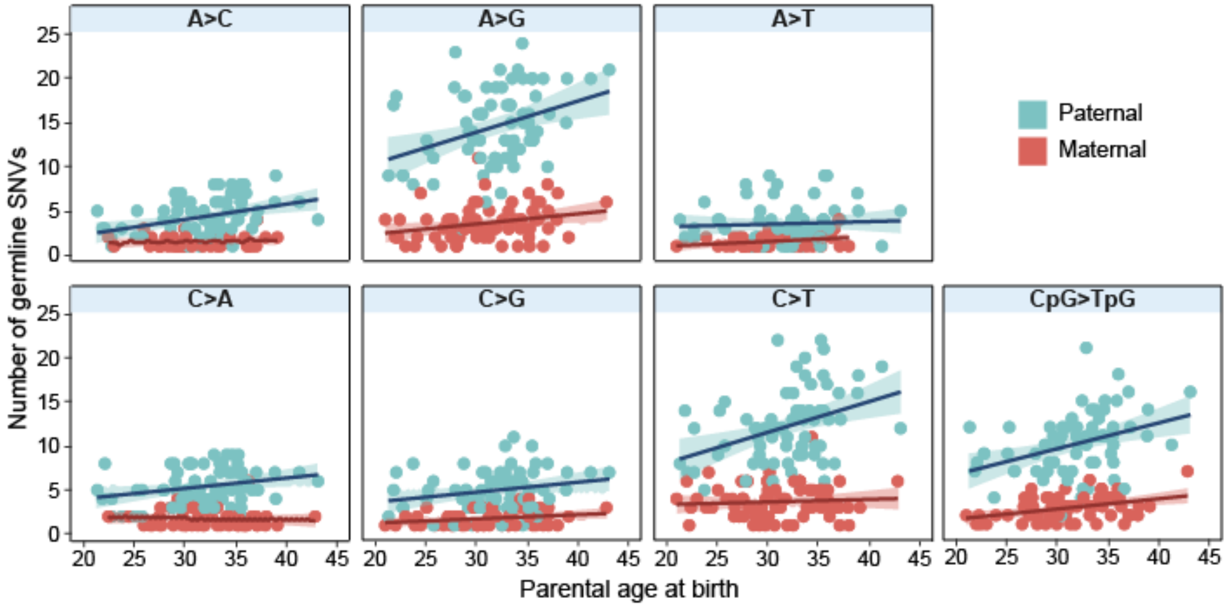

Figure S4: Parental age effect by mutation class
The number of paternal A>C, A>G, C>A, and CpG>TpG mutations are all significantly correlated with paternal age. For maternal mutations, there is a significant maternal age effect on A>G and CpG>TpG mutations.

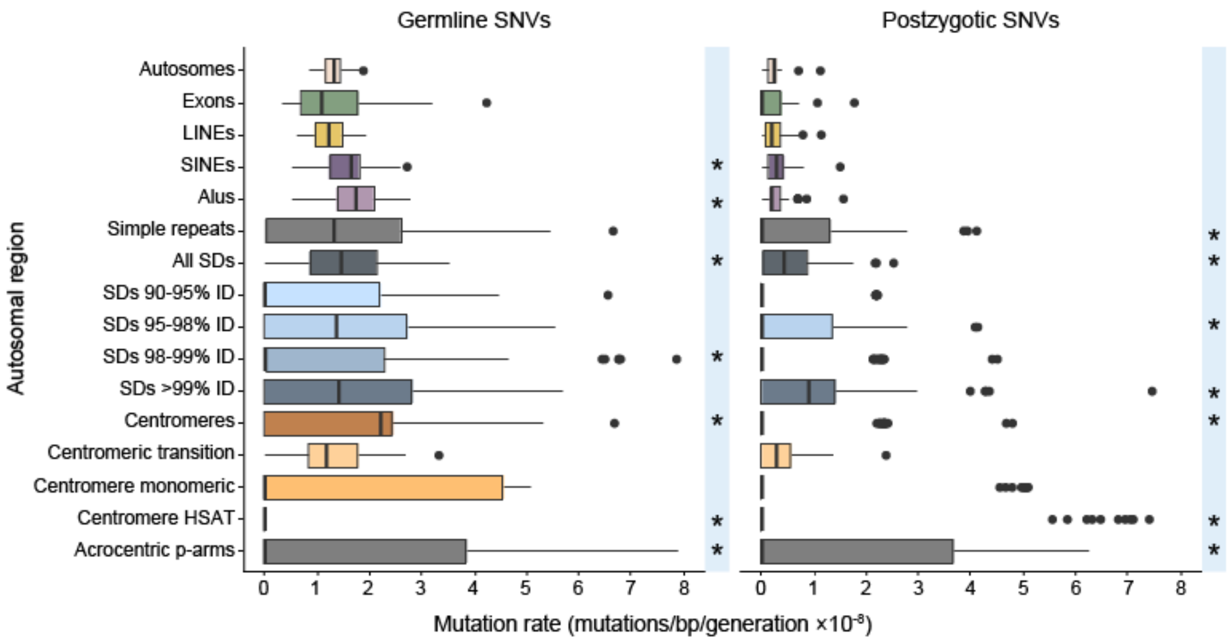

Figure S5: Mutation rates in segmental duplications and centromeric regions

The germline and postzygotic mutation rates are significantly enriched in the highest-identity segmental duplications and acrocentric p-arms.
